# Modeling of Soft Pneumatic Actuators with Different Orientation Angles Using Echo State Networks for Irregular Time Series Data

**DOI:** 10.3390/mi13020216

**Published:** 2022-01-29

**Authors:** Samuel M. Youssef, MennaAllah Soliman, Mahmood A. Saleh, Mostafa A. Mousa, Mahmoud Elsamanty, Ahmed G. Radwan

**Affiliations:** 1 Smart Engineering Systems Research Center (SESC), Nile University, Sheikh Zayed City 12588, Egypt; melsamanty@nu.edu.eg; 2 School of Engineering and Applied Sciences, Nile University, Sheikh Zayed City 12588, Egypt; msoliman@nu.edu.eg (M.S.); mabdullah@nu.edu.eg (M.A.S.); 3 Nile University’s Innovation Hub, Nile University, Sheikh Zayed City 12588, Egypt; mabdelrahman@nu.edu.eg; 4 Mechanical Department, Faculty of Engineering at Shoubra, Benha University, Cairo 11672, Egypt; 5 Nanoelectronics Integrated Systems Center (NISC), Nile University, Sheikh Zayed City 12588, Egypt; agradwan@nu.edu.eg; 6 Department of Engineering Mathematics and Physics, Cairo University, Giza 12613, Egypt

**Keywords:** Echo State Network (ESN), reservoir computing, Recurrent Neural Network (RNN), Long Short-Term Memory (LSTM), soft robotics, Soft Pneumatic Actuators (SPA), modeling

## Abstract

Modeling of soft robotics systems proves to be an extremely difficult task, due to the large deformation of the soft materials used to make such robots. Reliable and accurate models are necessary for the control task of these soft robots. In this paper, a data-driven approach using machine learning is presented to model the kinematics of Soft Pneumatic Actuators (SPAs). An Echo State Network (ESN) architecture is used to predict the SPA’s tip position in 3 axes. Initially, data from actual 3D printed SPAs is obtained to build a training dataset for the network. Irregular-intervals pressure inputs are used to drive the SPA in different actuation sequences. The network is then iteratively trained and optimized. The demonstrated method is shown to successfully model the complex non-linear behavior of the SPA, using only the control input without any feedback sensory data as additional input to the network. In addition, the ability of the network to estimate the kinematics of SPAs with different orientation angles θ is achieved. The ESN is compared to a Long Short-Term Memory (LSTM) network that is trained on the interpolated experimental data. Both networks are then tested on Finite Element Analysis (FEA) data for other θ angle SPAs not included in the training data. This methodology could offer a general approach to modeling SPAs with varying design parameters.

## 1. Introduction

The use of soft materials has recently gained traction in robotics and the field of soft robotics has seen many developments. The main reason for this is the advantages gained by using soft materials instead of building completely rigid robots, the main advantage being the high flexibility that could be achieved by soft robots. Building soft robots would allow us to mimic different living creatures and organisms with high robustness and adaptability to their environment. Moreover, soft robots are safer to interact and collaborate with humans than traditional robots with rigid bodies [1].

However, one of the main hurdles of building soft robots is the complex non-linear dynamics they exhibit due to the compliant nature of the soft materials used to build them [2]. These complex dynamics are hard to model, and subsequently are very challenging to control. Achieving high-level control of soft robots would allow them to be deployed in actual working environments to perform complex and intelligent tasks. Despite the challenges presented in building soft robotics systems, the advantages they offer over rigid robots are essential to achieve more complex and robust robotics tasks and reach high human-robot interaction. The limitations of rigid robots are mainly due to their designs using hard links and structures that have limited degrees of freedom, which are much easier to model and control using traditional algorithms. However, these robots are not safe to directly interact with humans in work environments, as well as not being robust enough to adapt to different environments and perform highly complex tasks. Consequently, many researchers have been concentrating on solving the modeling and control problems of soft robotics systems.

## 2. Related Work

Several approaches have been investigated to achieve modeling and control for soft robots. Some methods are based on mathematical analysis approximation of the soft structure such as the piecewise constant curvature model approximation [3], the geometrical exact approach such as the Cosserat rod theory [4], and the variable-strain method that generalizes the piecewise constant-strain approach [5]. Other methods rely on data-driven techniques such as neural networks and reinforcement learning [6].

However, the elastic behavior of the soft material leads to large deformation in the body of soft robots. Hence, it becomes extremely difficult to reach a general model for such robotic systems. Many attempts have been made in this area to model the deformation of soft actuators. Finite-element methods have been used by Moseley et al. to predict the displacement and force of a soft pneumatic actuator (SPA) [7]. Several attempts also investigated the modeling of a fiber-reinforced soft actuator using continuum models [8,9,10]. Another model that’s widely used is the Euler-Cantilever-Beam model, which assumes that the soft actuator behaves like a cantilever beam [11,12,13]. Other approaches tried to split the actuator into several small segments and study the bending of each segment separately, then add them together to estimate the total bending of the whole actuator [14].

Furthermore, several groups have been investigating data-driven approaches. Most notably, the use of different machine learning and deep learning models is showing promising results. These models rely on training neural networks that are capable of predicting the deformation of the soft material and the position of the actuator tip or end-effector. Some proposed models are linear regression models [15]. One approach used simulation data from a Finite Element Method (FEM) hyperelastic material model to train an Artificial Neural Network (ANN) to predict the bending angles of SPAs with variable geometrical parameters [16]. However, the most commonly used networks to model the time series data obtained from the actuator are Recurrent Neural Networks (RNNs). Thuruthel et al. embedded soft sensors into the actuator to obtain bending data and used it to train an RNN that can predict the position of the actuator’s tip and the force applied by it [17]. Another group used a Bidirectional Long Short-Term Memory (BiLSTM) network, a type of RNN, to estimate the position of a hydraulic soft hybrid sensor-actuator [18].

Despite offering reasonable accuracy, RNNs still struggle to fully map the input-output relationship of the soft actuator. ESNs provide a possible solution to this problem, due to their ability to model the non-linear dynamics of complex systems. They depend on the concept of Dynamic Reservoir (DR) computing and they tend to simulate the actual soft robotic system more accurately, due to their closeness to the real system. Some attempts have been made to use ESN to model and control complex dynamic systems, such as the CoroBot’s Arm [19]. Sakurai et al. also used an ESN to model a McKibben Pneumatic Artificial Muscle (PAM) [20].

**Figure 1:**
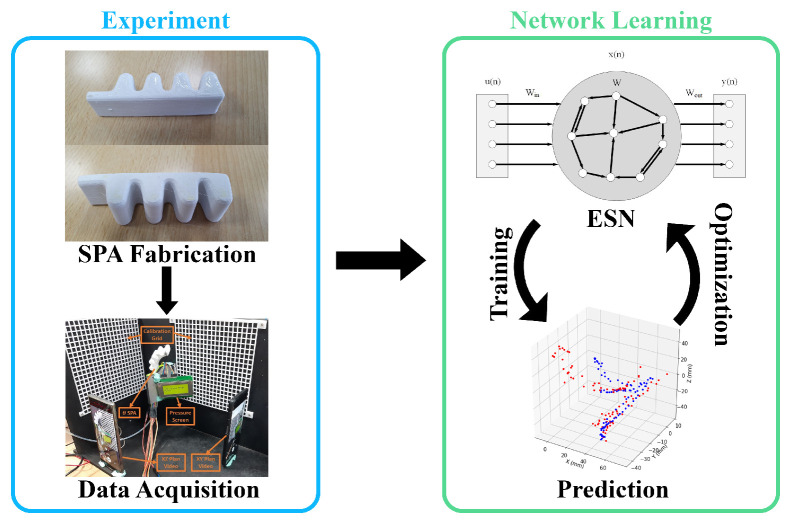
The pipeline for the modeling of the SPA using ESN. Ground truth data is obtained for different input actuation sequences of the SPA (**left**). The ESN learning process (**right**) is done in a cyclic manner. The network is first trained with fixed parameters, then the output weights are fixed and the parameters are optimized. The process is done iteratively till convergence.

In this article, an ESN is used to model an SPA using irregular data, and to predict the position of the actuator’s tip in 3 dimensions (Figure 1). In the next section, an overview of the SPA used in the experiment is presented, including its design features. The concept behind ESNs is also discussed. In the subsequent sections, the experiment conducted is demonstrated in detail with the ESN training, and the results attained, showing the performance of the network in predicting the SPA tip position in 3D. In addition, a Long Short-Term Memory (LSTM) network is trained on the interpolated data and its performance is compared against the ESN’s. Finally, both trained networks are tested for their ability to generalize using data obtained from Finite Element Analysis (FEA) simulation.

## 3. Methodology

The proposed approach is to use machine learning techniques that can emulate the non-linear dynamics of complex systems to predict the 3D position of the tip of the Soft Pneumatic Actuator (SPA) due to the deflection of the soft material after applying pressure to the actuator. One class of neural networks suitable for such spatio-temporal task is the RNN, as it possesses internal states that act as memory. In this experiment, we used the ESN, which is based on the concept of Reservoir Computing (RC), and compared its results with the LSTM network, which is one of the most promising networks used with time series data. This section presents the details of the SPA used, the experiment performed including the data acquisition process, and the models used to predict the SPA’s kinematics.

### 3.1. Soft Pneumatic Actuator (SPA)

SPAs, widely known as Pneunets, could be used to develop soft robots that can achieve complex movements and locomotion. In general, SPAs have air chambers that allow the soft material to contract and expand when pneumatic pressure is applied to their internal structure. Applying different pressure values, whether positive or negative pressure, makes the actuator reach different positions in 3D space. Several design parameters also affect the performance of the SPA, such as the geometry of the air chambers and their number, the orientation and inclination angles of the chambers, the length of the actuator, and its thickness. All these factors, in addition to the non-linear nature of the soft material, contribute to the complexity of modeling of the SPA. Conventional modeling methods used to describe rigid robots’ kinematics cannot be applied in the case of soft robots. Instead, a common method used to estimate the behavior of SPAs is the FEA, which could simulate the deformation of the hyperelastic material under different operating values. However, this process requires intensive computation power and is very lengthy. New research proposes the use of differentiable simulation as a method of modeling soft robots and bridging the simulation-to-reality gap [21].

**Figure 2:**
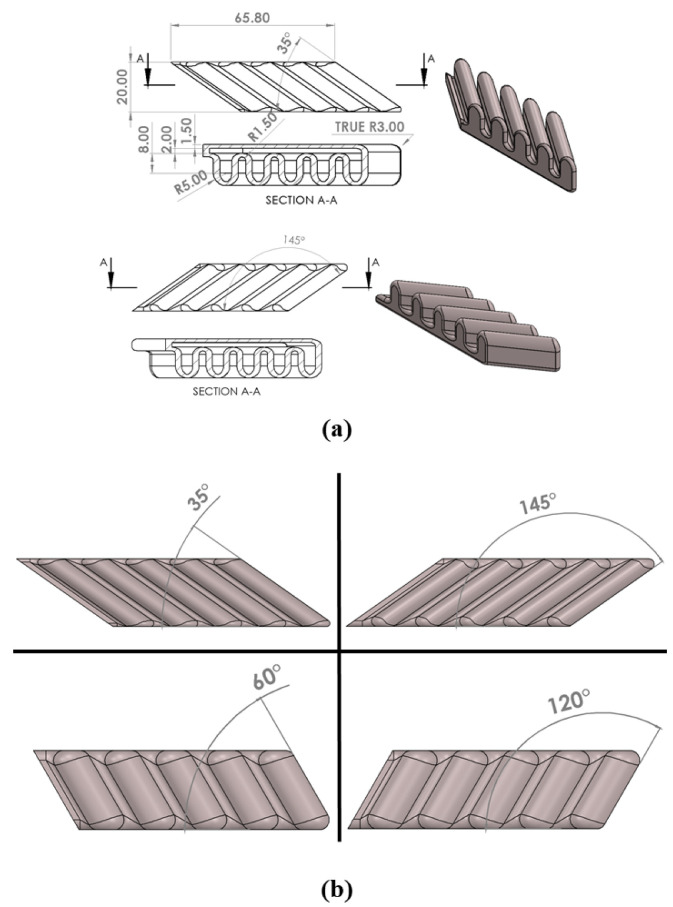
3D Design and geometrical dimensions of the SPA for orientation angles bigger and smaller than 90${}^{\circ }$. (**a**) Detailed CAD design with dimensions. (**b**) SPAs θ = [$35^{\circ }$, $60^{\circ }$, $120^{\circ }$, $145^{\circ }$] demonstration that shows the orientation angle effect.

Previous work has discussed the process of soft material identification, and the design, manufacturing, FEA simulation, and validation for SPAs of different inclination angles [22]. The effect of varying the inclination angle of the chambers on the gripping force was investigated. The yielding and buckling issues of the SPA’s bending and their effects on the output force are also discussed in [23]. In [24], 4 SPAs (seen in Figure 2) with different orientation angles of 35${}^{\circ }$, 60${}^{\circ }$, 120${}^{\circ }$, and 145${}^{\circ }$ were fabricated using Fused Deposition Modeling (FDM) 3D printing with the Felix Tec 4. In addition, CURA software is used for slicing the CAD design. The SPAs are made from Thermoplastic Polyurethane (TPU) soft material, which require certain printing parameters such as 0.1 mm layer height, 0.4 mm line width, and a temperature of 220 ${}^{\circ }$C. To improve the sealing, the final layer is heated using the ironing feature. The infill density of the actuators is 100% and the adhesion between the layers was improved by increasing the contact area between the side walls and top layers using inner fillets. The SPA orientation during printing was set in the pull direction of the extrusion nozzle. No support materials were used. The SPAs were tested by applying pressure and observing the deflection of the SPA’s tip in 3D space. The SPAs were used as the flippers for a biomimetic turtle robot and its motion was analyzed. In this current work, an ESN is used to model these SPAs and predict the tip position within its 3D work envelope.

### 3.2. Experiment

A practical experiment is conducted on 3D-printed SPAs to acquire training and testing data for the ESN and assess the performance and accuracy of the proposed method.

#### 3.2.1. Setup

**Figure 3:**
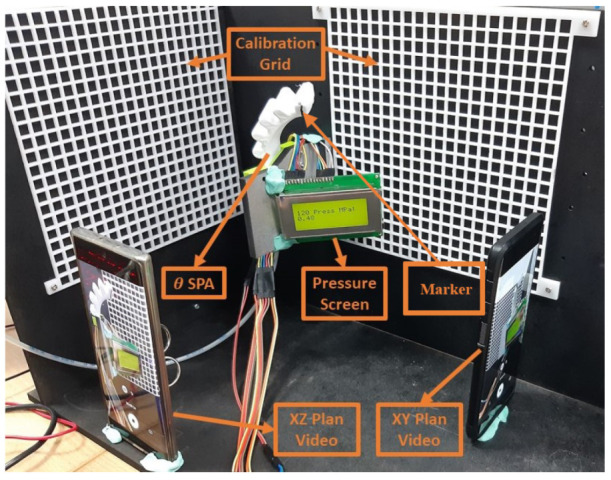
Experimental setup for the actuation of the SPA, including the calibration grid, cameras, and pressure visualization for data acquisition.

The experiment’s setup, as shown in Figure 3, is mainly comprised of the 3D-printed SPA, which is subjected to a varying operating pressure, and a vision system acting as a feedback method to capture the ground truth data used to train the ESN. The SPAs were 3D-printed with 4 different orientation angles of 35${}^{\circ }$, 60${}^{\circ }$, 120${}^{\circ }$, and 145${}^{\circ }$. Several trials were conducted on each SPA by applying pneumatic pressure to actuate the SPA using compressed air. A high-speed valve was controlled using pulse width modulation (PWM) to vary the input pressure inside the SPA from a range of 0.1 to 0.6 Megapascal (MPa). During each trial, the SPA was driven by a sequence of actuation pressures and its behavior was observed. The 3D position of the actuator’s tip is obtained by analyzing the recorded video stream of the trials, using computer vision tracking software. The Tracker video analysis tool is built upon the Open Source Physics library [25].

#### 3.2.2. Data Acquisition

The acquisition of the ground truth data for the training procedure was done using two cameras. The actuation trials were recorded from two views: an *x*-*y* plane, and an *x*-*z* plane, to obtain the actuator’s motion in the 3 dimensions. Markers were placed on the actuator’s tip to enable position tracking during the image processing stage (seen in Figure 3). The two streams were calibrated using a calibration grid and synchronized to obtain the 3D position at each actuation step. Finally, the Tracker tool was used to annotate the videos and extract the ground truth positions, as described in [24]. The tool auto tracks the pixels containing the marker on the actuator’s tip and extracts its coordinates for each frame of the video.

During the data acquisition, the main focus was to capture the final position for each actuation step. Due to the non-linear dynamics exhibited by the SPA, the transient time varies between the different actuation pressures. In addition, the memory effect exhibited by the SPA affects the dependence of the state of the actuator on previous actuation forces. To capture such behavior, the pressure sequences used during the experiment consist of pressure steps sampled at irregular intervals between them, to showcase the response of the SPA to the same pressure input but coming from different previous input pressures. For this reason, the recorded data is represented in irregular time steps. The data is used to train both the ESN and LSTM models.

### 3.3. Modeling Using Echo State Network (ESN)

The ESN is a special type of RNN. It was first proposed by H. Jaeger [26]. It is one of two paradigms, the other being Liquid State Machines, that are known as reservoir computing (RC) [27]. The reservoir computing concept is inspired by recurrent neural networks. It is based on creating a complex dynamical system (reservoir) with non-trainable weights. The input signals are connected to this reservoir via fixed weights, thus, allowing the mapping to higher dimensional computational spaces (embedding). The embedding could then be connected to the output via a simple linear trainable layer (readout layer).

**Figure 4:**
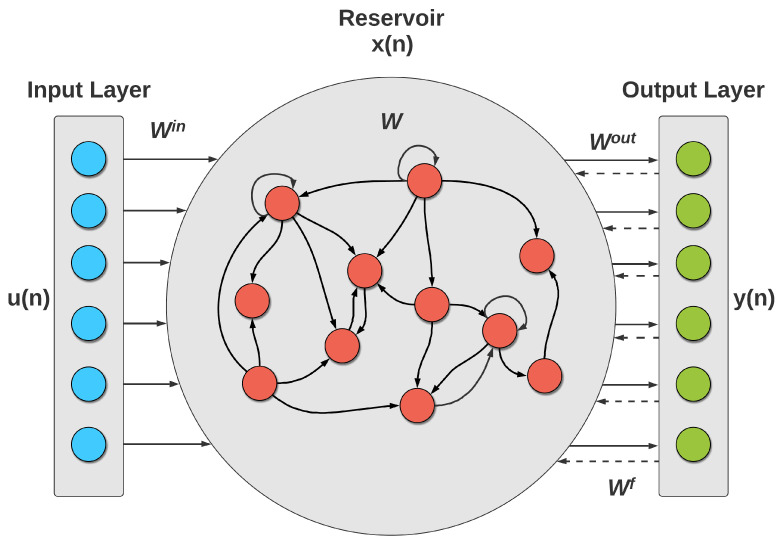
ESN Architecture, having a time series input, a reservoir of neurons with a specified activation function and a linear output layer.

The main component of the ESN is the dynamic reservoir that constitutes the RNN’s hidden layer. The ESN’s ability to model complex dynamic systems comes from the complex structure of the reservoir. That’s why the reservoir needs to contain a large number of neurons that are excited to map the input to the output [28]. The architecture of the ESN is shown in Figure 4.

The hidden layer mainly constitutes a large number of hidden neurons that are sparsely connected via fixed and randomly assigned weights. The only trainable weights in the network are those of the output neurons. These weights transform the non-linear dynamic behavior of the reservoir into a linear output that could be learned to reproduce a system’s spatio-temporal response. An output feedback weight matrix could also be included and are generated with the system states through teacher forcing using the correct outputs.

A basic ESN consists of *N* reservoir units, *U* input signals, and *L* outputs. The state update equations are:

(1) $$\tilde{x}\left(n\right)=f\left(W^{in}u\left(n\right)+Wx\left(n-1\right)+W^{fb}y\left(n-1\right)\right)$$

(2) $$x\left(n\right)=\left(1-\alpha \right)x\left(n-1\right)+\alpha \tilde{x}\left(n\right)$$

where x(n) is the reservoir state of *N* dimensions and $\tilde{x}\left(n\right)$ is its update, *f* is the activation function of the reservoir neurons, u(n) is the input signal of *U* dimensions, *W* is the N×N reservoir weight matrix, $W^{in}$ is the N×U input weight matrix, y(n) is the output signal of *L* dimensions, and $W^{fb}$ is the N×L output feedback weight matrix, which can be eliminated. α is the leaking rate of the neurons.

The output is obtained through the linear readout layer using the equation:

(3) $$y\left(n\right)=f^{out}\left(W^{out}\left[u\left(n\right);x\left(n\right)\right]\right)$$

where $f^{out}$ is the output activation function and $W^{out}$ is the L×(U+N) output weight matrix that the network is required to learn to get the desired output. [u(n);x(n)]) is the extended system state.

Using the above equations, the ESN can be trained to learn a spatio-temporal model with the desired output signal $y^{target}\left(n\right)$ of *L* dimensions. The training part could be treated as a supervised machine learning task, to minimize a loss function such as the Root-Mean-Square Error (RMSE) equation described here:

(4) $$E\left(y,y^{target}\right)=\frac{1}{L}\sum_{i=1}^{L}\sqrt{\frac{1}{T}\sum_{i=1}^{T}{\left(y_{i}\left(n\right)-y_{i}^{target}\left(n\right)\right)}^{2}}$$

where *T* is the number of training data points. The training dataset can have sequences of different lengths [29].

#### 3.3.1. Network Architecture

The ESN architecture mainly comprises a certain number of neurons in the reservoir. The network consists of three types of connection weights. The first type is the connection weights from the input nodes to the reservoir neurons ($W_{in}$). The second type is the internal connection weights of the reservoir neurons (*W*). The last type is the connection weights from the reservoir neurons to the output nodes ($W_{out}$). Connection weights $W_{in}$ are randomly generated real values and are fixed throughout the experiments. The network also has important parameters that affect its learning ability, mainly the spectral radius of the reservoir weight matrix. It affects the decay time of the input impulse response and the non-linear interaction of inputs. One other parameter is the sparsity or the connectivity of the reservoir weight matrix. It determines the number of connections between the reservoir neurons, which affects the dynamics of the reservoir and the variation of its signals. Higher sparsity makes the reservoir act as loosely coupled subsystems [26], which helps capture the dynamics of the system being modeled. The activation function for the reservoir neurons is also an important parameter, with the most commonly used being tanh.

For this experiment, the input of the network is the applied pressure and the orientation angle (θ) of the SPA, and the output to be predicted are the *x*, *y*, and *z* positions of the actuator’s tip. The trials were split into training and testing subsets. A total of 52 actuation sequences were used with a total of 1128 actuation steps, divided into 36 and 16 sequences (794 and 334 steps) for training and testing, respectively (70–30% train-test split). Pre-processing on the data is done by converting (θ) from degrees to radians, and converting the positions from millimeter (mm) to meter (m).

The training is performed in two stages. First, the network is trained with fixed hyperparameters, only training the output weights. Next, a Bayesian optimization method is used to reach the best parameters for the network. During the optimization training, the weights of the output layer are fixed from the previous training, while the network hyperparameters are optimized. Iteratively alternating between these two steps allow us to reach the optimized hyperparameters and output weights. All codes were implemented in Python.

#### 3.3.2. Results

By training the ESN on the data obtained from the experiment, The network can predict the 3D position of the actuator’s tip with reasonable accuracy. Several training experiments were conducted with different network parameters.

At first, the basic implementation of the ESN is used to train a network with different reservoir sizes ranging from 1000 to 10,000 neurons. The network is trained to predict the tip’s position in the three axes *x*, *y*, and *z* simultaneously. Other network parameters were fixed, with a spectral radius of 0.95, a sparsity of 0.97, a regularization of 0.1, and a random seed of 42 for the initialization of the non-trainable weights. The network with 10,000 neurons achieved a training error of $1.85\times 10^{-13}$ and a testing error of 11.

However, the predictions for some test sequences, particularly for the *x* position, are less accurate. Thus, to improve the results, the network’s parameters need to be optimized. To achieve this, the Bayesian optimization approach described in [30] is used to optimize 7 parameters: reservoir size, input scaling, feedback scaling, leaking rate, spectral radius, sparsity, and regularization.

**Figure 5:**
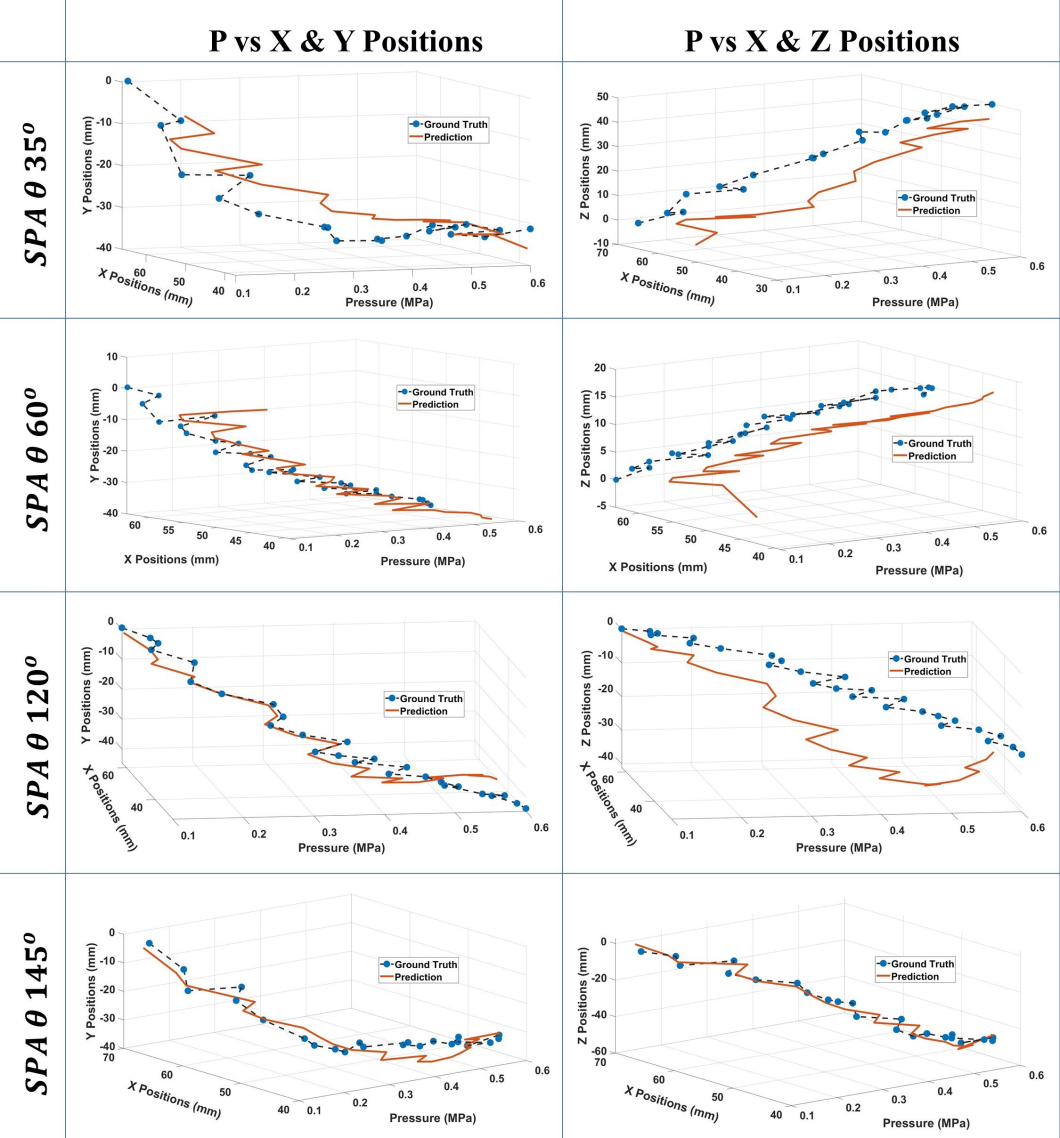
Prediction results of the ESN network after optimizing the 8 hyperparameters. 4 test samples, one from each θ angle, are plotted. The plots show the network’s predictions for the SPA tip’s motion plotted against the input pressure.

In addition, to achieve more accurate results for each position, the training and optimization process is performed on each axis individually, to get three separate sets of optimized parameters, one for each of the 3D positions, as seen in Table 1. In comparison, the network achieved improved testing errors than the network with manually set hyperparameters. The errors are 5.72162, 4.63309, and 15.74720 for *x*, *y*, and *z*, respectively. The network’s results are shown in Figure 5.

**Figure 6:**
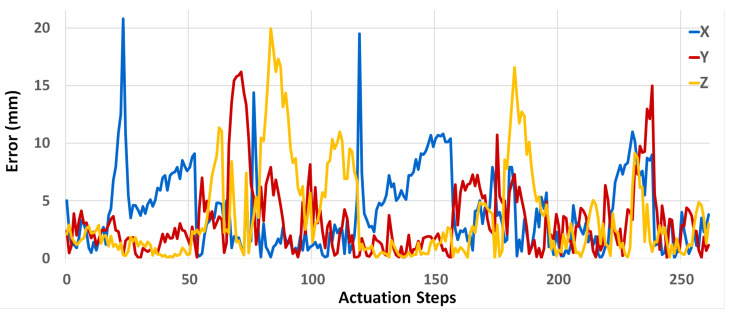
Prediction errors for optimized ESN. The error plot of the test sequences for the 3 motion axes *x*, *y*, and *z*.

By inspecting the network’s error, some outlier results can be observed in three test sequences, which were omitted from the testing subset. The prediction error plot and error range are shown in Figure 6. The maximum prediction error happens in the *x*-axis, up to 22 mm. For the *y* and *z* axes, the maximum error is about 16 and 20 mm, respectively. The average error for the 3 axes ranges between 3 and 4 mm.

Despite the improvement in the accuracy, the model still exhibits high prediction error for some test sequences, specifically in the *x*-axis. This could be due to several factors, such as the small displacement happening in the *x* direction compared to the *y* and *z* directions, and the nature of the SPA system such as its hysteresis effect, causing its current position to depend on the past inputs and the same output position could be reached through more than one specific input sequence. This is due to the elastic property of the soft material, which is hard for the network to fully map. However, the ESN can capture the complex non-linear dynamic behavior of the soft actuator by relying only on the actuation input and without any sensory feedback, even when varying design parameters such as the orientation angle in this case.

### 3.4. Modeling Using LSTM

The LSTM is another type of RNN that is prominently used for time series prediction [31]. To compare the ESN performance, we train an LSTM network on the SPA data. However, the temporal irregularity of the data affects the ability of these types of networks to model the behavior of the system [32]. Two trials were conducted. The first trial used the irregular data to train an LSTM network consisting of 6 hidden layers with sizes of 512, 256, 128, 64, 32, and 8 units, respectively. The network’s performance is the same as the ESN network. Despite this, the training requires significantly more time and computational resources than the ESN, and the model is prone to overfitting, due to the large number of parameters to train by comparison with the small dataset used.

**Figure 7:**
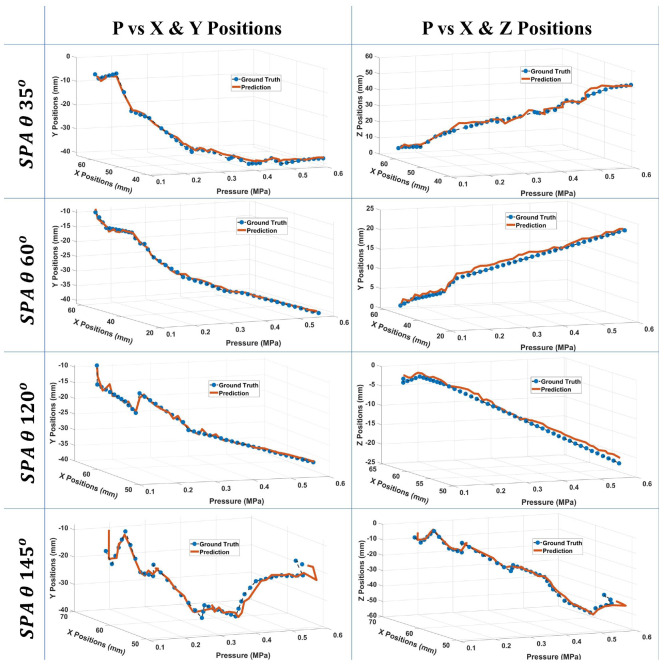
Prediction results of the LSTM network trained on the interpolated data. The plots are for 4 test samples.

**Figure 8:**
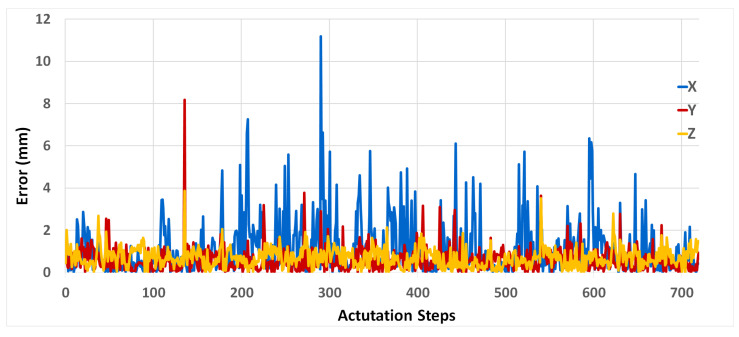
Prediction errors for the LSTM on the interpolated data. The error plot of the test sequences for the 3 motion axes *x*, *y*, and *z*.

In the second trial, pre-processing on the data is done to handle the irregularity. To overcome this, one common approach is the use of an imputation method such as data interpolation. The SPA data obtained from the experiment are interpolated to estimate the intermediate values between the observed time steps. The LSTM architecture used consists of a hidden layer with 512 units (Table 2). A fully connected layer provides the final output. The Adam method is used to optimize the network weights. In addition to the pre-processing previously done on the data, the values were scaled using min-max normalization. The input data is transformed using the sliding window approach with a look-back value of 5, to include the input and output data of the previous time steps alongside the inputs for the current time step. The LSTM performance on the test set achieved an RMSE of 1.66453, 0.83381, and 0.83234 for *x*, *y*, and *z*, respectively. The results for four test samples, one for each SPA orientation angle, are shown in Figure 7. The errors plots (Figure 8) show a maximum error of 11 mm in the *x* position.

### 3.5. Model Validation Using FEA Data

In order to test the models’ ability to learn the general kinematic behavior of the SPA, The two trained networks are tested using data from SPAs with 22 different θ angles, including data for the 4 SPA angles previously used for training, as well as, 18 other orientation angles, ranging from 35${}^{\circ }$ to 145${}^{\circ }$, with an interval of 5${}^{\circ }$. However, due to the inability to fabricate all the SPAs and perform the experiment on them, the FEA data is used instead for this validation process. The FEA data was obtained using Ansys Multi-Physics 2019 R2^TM^. The TPU material is identified using the Mooney-Rilivin five parameters material hyperelastic model and applied to the 22 SPAs with different θ angles [24].

**Figure 9:**
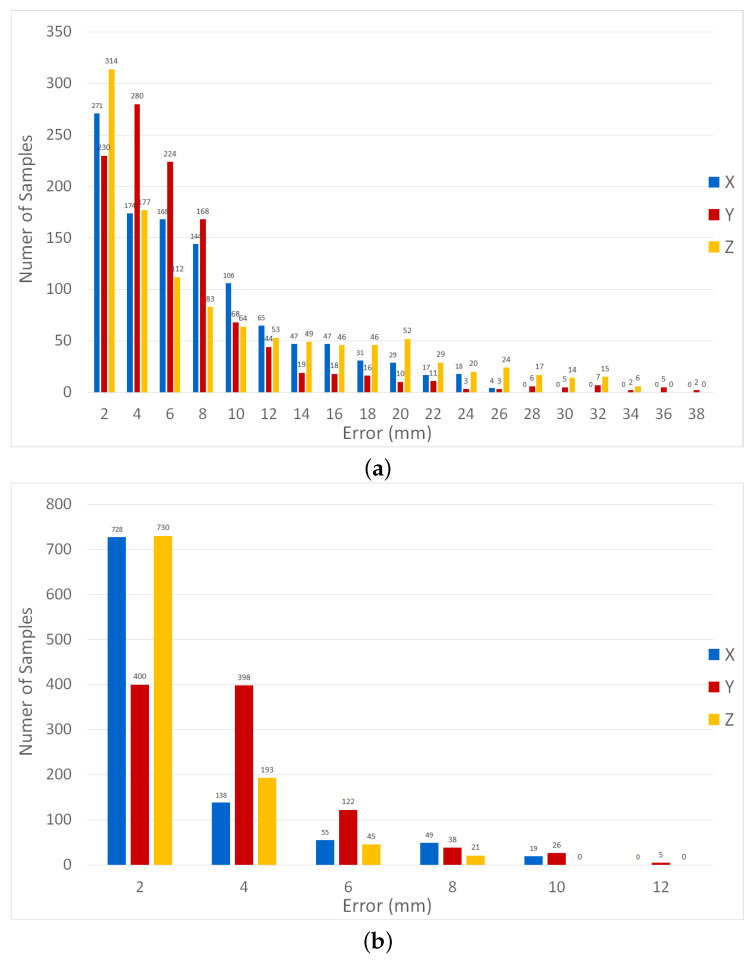
Histogram of the prediction errors on the FEA data. (**a**) ESN predictions. (**b**) LSTM predictions.

The FEA data is then used to test the ESN and the LSTM performance. The ESN achieved an RMSE of 8.76696, 8.25630, and 11.69436 for *x*, *y*, *z*, respectively. By comparison, the LSTM errors are 2.5964, 3.3739, and 2.0618. The prediction results are illustrated in the Supplementary Materials. The errors histograms from both the ESN and the LSTM are presented in Figure 9.

The FEA data is then used to test the ESN and the LSTM performance. The ESN achieved an RMSE of 8.76696, 8.25630, and 11.69436 for *x*, *y*, *z*, respectively. By comparison, the LSTM errors are 2.5964, 3.3739, and 2.0618. The prediction results are illustrated in the Supplementary Materials. The errors histograms from both the ESN and the LSTM are presented in Figure 9.

## 4. Discussion

Deriving an accurate mathematical model for robots or actuators made from soft materials is hard to achieve using previously known methods such as the piecewise constant curvature. Due to the dynamic complexity of such models, the use of data-driven approaches is suitable to map the input-output relations of these systems and make predictions.

The approach proposed in this study is to use an ESN to model the kinematics of SPAs with different orientation angles θ. By gathering experimental data from SPAs and training the ESN to predict the position of the SPA’s tip, the network prediction shows promising results in the ability of the ESN to model soft robotic systems with non-linear complexity. In the first training phase, the ESN is trained with fixed hyperparameters, which leads to poor results. In the second phase, the hyperparameters are optimized using the Bayesian technique to achieve more accurate predictions. Increasing the training dataset with more SPA actuation sequences could potentially improve the network’s accuracy.

RNNs are generally used in modeling tasks that require learning spatio-temporal behavior of complex systems, due to the memory units they contain. In the case of SPAs, the actuator has a physical memory that exhibits hysteresis. Its position is affected by the previous actuation forces applied on it and the large deformations caused by them. Even after removing the actuation pressure, the SPA might not return to its initial state. The properties of the soft TPU material of the SPA contribute to this behavior, alongside its geometrical parameters. The RNN’s ability to deal with memory makes it a suitable choice for this modeling task.

In the proposed methodology, the use of the ESN architecture is justified by its reliance on the concept of reservoir computing. It offers the ability to represent the high-dimensional dynamic system of the SPA as a reservoir with a small number of internal neurons. It is easier to train using a small dataset and computing the output weights of the linear readout layer by simply using linear regression. By comparison, other RNN architectures would require a large amount of data to exploit the gradient descent method used to train their large number of weights to obtain accurate models. In addition, the process of collecting a large amount of data from these types of soft systems would cause their physical properties and inherent behavior to be altered significantly. It would also shorten their lifespan, which is undesirable in practical settings. Thus, comes the need for the high efficiency of the data-driven modeling technique and the small amount of data required to adjust the network’s parameters to adapt to the changes happening in the soft system over time.

In addition, the task of proprioceptive sensing for soft robots is a challenging issue. The embedding of sensory devices within the soft materials could undesirably affect their deformability. The presented approach doesn’t include any embedded sensors in the SPA. This requires the used network architecture to be able to learn the system’s behavior using only the low-dimensional input feature, being the input pressure force in this case.

One of the challenges for this type of experiment is the data acquisition method. The non-linear nature of the SPA, its soft material memory effects, and the restriction on the amount of data to collect without damaging the system are taken into account. This leads to irregular actuation steps as discussed in the data acquisition section. To investigate the performance of other RNN architectures such as the LSTM and compare it to the ESN’s, a pre-processing interpolation method needs to be applied to the data, as the irregularity of the data would affect the LSTM’s ability to accurately learn the kinematics model. However, the linear interpolation estimation of the intermediate (in-between) actuation steps is not accurately representative of the actual physical system. By comparison to the ESN, the LSTM also requires a longer training time. The total number of parameters needed for the LSTM training is 1,085,953, compared to only 2067 parameters for the ESN training for the *x* position, which is the largest reservoir size.

Our methodology also tests the ability of the models to generalize and predict the kinematics of SPAs with different θ angles that were not included in the training data. However, due to fabrication limitations, the data used to test the networks is obtained from the FEA, which doesn’t account for the actual behavior of the system due to the lack of biaxial and shear test for the TPU material’s hyperelastic model as illustrated in [24], but it provides a close estimation for the bending behavior trend. The test errors for both the ESN and LSTM networks on the experimental and FEA data are presented in Table 3. It would be beneficial to investigate more network architectures and collect more experimental data from various SPAs. A real-time end-to-end model capable of adapting to the system’s changes over time would require an accurate real-time visual sensing system and continuous online training.

## Figures and Tables

**Table 1:** Optimized Parameters of ESN for the 3 individual axes.

Parameter	X	Y	Z
Reservoir Size	2065	100	100
Input Scaling	1	0.646784	1
Feedback Scaling	1	0.30386	1
Leaking Rate	1	1	1
Spectral Radius	0.5	0.550113	0.855145
Sparsity	0.999	0.999	0.999
Regularization	10	0.07043	0.029732
Random Seed	123	123	123
Trainable Output Weights	2067	102	102

**Table 2:** LSTM network parameters.

Parameter	Value
Hidden Layers	1
Recurrent Units	512
Look-back	5
Optimizer	Adam
Data Scaling	Min-max Normalization
Learning Rate	0.01
Batch Size	1
Epochs	500
Trainable Weights	1,085,953

**Table 3:** Test errors for ESN and LSTM networks on experimental and FEA data.

Test	X	Y	Z
ESN on Experimental Data	5.72162	4.63309	15.74720
LSTM on Experimental Data	1.66453	0.83381	0.83234
ESN on FEA Data	8.76696	8.25630	11.69436
LSTM on FEA Data	2.5964	3.3739	2.0618

## Supplementary Materials

The following are available online at https://www.mdpi.com/article/10.3390/mi13020216/s1.
